# Age at Sexual Debut and High-Risk Sexual Behavior in Association to HPV Risk in Two Regions of Peru

**DOI:** 10.51894/001c.164054

**Published:** 2026-07-31

**Authors:** Elizabeth Lossada-Soto, Giovanna Russano, Elizabeth Macdonald, Ruben Briceno

**Affiliations:** 1 College of Osteopathic Medicine, Michigan State University

## 28

### Background

Cervical cancer is the leading cause of malignant neoplasms and the second leading cause of death for Peruvian women. To better serve Peruvian women, this study investigates two risk factors for the root cause of cervical cancer in Peru and worldwide – human papillomavirus (HPV) infection.

### Objectives

This study investigates how significantly an early age of sexual debut can change the likelihood of contracting HPV. The study also examined whether the number of lifetime sexual partners increases HPV risk and whether this factor correlates with the age of sexual debut.

### Methods

In 2024, cervical cells samples were collected from two distinct regions (and ethnicities) of Peru. This cross-sectional study enrolled 229 women from La Libertad (n=115) and Loreto (n=114), who presented for cervical screenings. All samples were analyzed using PCR along with identifying HPV presence and its genotype. A validated questionnaire was used to collect data. Analysis was done in SAS, utilizing both multivariate and Firth’s logistic regression.

### Results

Women whose age of sexual debut was <14 years old, the odds of HPV+ are 3.88 (95% CI= 1.16, 12.96) times higher among those with >=5 lifetime partners compared to those with <5 lifetime partners. For women whose age of sexual debut was >=14 years old, the odds of HPV+ are 38.29 (95% CI=11.28-130.01) times higher among those with >=5 lifetime partners compared to those with <5 lifetime partners. The association between lifetime number of sexual partners and HPV positivity is statistically significant (adjusted model interaction *p* -value= 0.0099). Both >=14 and <14 years old at sexual debut showed an increased risk of future HPV positivity if they had >=5 lifetime partners.

### Conclusions

Across both regions of Peru, our findings did not find that age of sexual debut was the most prominent variable in future HPV risk. Additionally, a higher number of sexual partners (>=5) is a more significant factor in future HPV positivity in this population over other factors such as consistency of condom use, STI history, and history of cervical screening, regardless of age of sexual debut.

